# Origin of symbol-using systems: speech, but not sign, without the semantic urge

**DOI:** 10.1098/rstb.2013.0303

**Published:** 2014-09-19

**Authors:** Martin I. Sereno

**Affiliations:** 1 Experimental Psychology, University College London, London, WC1H 0AP, UK; 2 Department of Psychological Sciences, Birkbeck College, University of London, London, WC1E 7HX, UK; 3 Birkbeck/UCL Neuroimaging Centre, 26 Bedford Way, London, WC1H 0AP, UK; 4 Cognitive Science Department, University of California, 9500 Gilman Drive, San Diego, La Jolla, CA 92093

**Keywords:** origin of language, sexual selection, code-use, scene comprehension, RNA world, protein folding

## Abstract

Natural language—spoken and signed—is a multichannel phenomenon, involving facial and body expression, and voice and visual intonation that is often used in the service of a social urge to communicate meaning. Given that iconicity seems easier and less abstract than making arbitrary connections between sound and meaning, iconicity and gesture have often been invoked in the origin of language alongside the urge to convey meaning. To get a fresh perspective, we critically distinguish the *origin* of a system capable of evolution from the subsequent *evolution* that system becomes capable of. Human language arose on a substrate of a system already capable of Darwinian evolution; the genetically supported uniquely human ability to learn a language reflects a key contact point between Darwinian evolution and language. Though implemented in brains generated by DNA symbols coding for protein meaning, the second higher-level symbol-using system of language now operates in a world mostly decoupled from Darwinian evolutionary constraints. Examination of Darwinian evolution of vocal learning in other animals suggests that the initial fixation of a key prerequisite to language into the human genome may actually have required initially *side-stepping* not only iconicity, but the urge to mean itself. If sign languages came later, they would not have faced this constraint.

## 1. Introduction

The origin of human language is intrinsically interesting to humans. This difference between humans and other sentient animals must have been obvious to Palaeolithic humans; and the recent resurgence of interest in language origins (e.g. [1–10]) has been little hindered by the paucity of hard evidence (a Pleistocene video would be nice)—or sensible admonitions to attend to more tractable problems. The following attempts to bring a fresh perspective by using an analogy with the origin and evolution of cellular coding systems. In other places [11–15], I have argued that DNA-and-protein-based life and language-based human thought may have enough in common—as the only two naturally occurring examples of a system using long code strings to construct thousands of parallel self-assembling meaning strings—to make it fruitful to use one system to make analogical predictions about the other. Two insights reached in the course of developing that analogy are useful here: (i) the difference between the *origin* and the *evolution* of a symbol-using system and (ii) the critical role played at the origin of the system by intermediate strings of ‘symbol-representation’ segments with properties partway between symbol and meaning. This unconventional starting point leads us to a new physical perspective on iconicity and arbitrariness.

Predictive analogy [16]—where a map of object properties and relations is drawn between a source and a target field to make predictions about less well-understood objects and relations in the target field—has served in the history of science as a strategy for nudging the mind off the unconscious rails on which it often runs within a field. For example, Darwin's *Origin of Species* is essentially a book-length analogy between animal breeding (artificial selection) and a postulated analogue of it in nature untouched by humans (natural selection). Merely wandering off track, however, does not help unless support for a resulting new idea can be adduced from within the target field. But analogical mapping can also provide insight into the source domain. Much of the present issue concerns an analogy between spoken language and signed language, which are implemented with different sensory and motor modalities and differ in their detailed structure and brain implementation. Our understanding of human language capabilities has been deepened and generalized by using that mapping in both directions. Certainly, the analogy with cellular-level coding systems spans a much greater distance then that one. But that very distance from uniquely human abilities promotes objectivity. Interestingly, the analogy between human and cellular symbol use was used by the first molecular biologists to reason about cells, not the other way around [13,17].

## 2. Origin versus evolution

In discussions about how life came into existence, it is common to distinguish the *origin* of life from the *Darwinian evolution* of life [18]. The core of every living cell is a system for converting genes into proteins—that is, DNA symbol sequences into amino acid meaning chains that spontaneously fold into the three-dimensional molecular machinery of the cell, which includes enzymes, receptors, fantastical diffusion-driven membrane rotors for generating ATP [19], force-producing strands that use ATP and so on. Though this is confusingly called ‘protein translation’ for historical reasons (based on the original molecular biologists’ casual and faulty analogy with Morse code!), it is now understood by molecular biologists as the process by which DNA symbol strings are turned into phenotypic protein meanings.

The problem of the *origin* of such a system, however, is not really an *evolutionary* problem in the usual Darwinian sense of the word. If we shrink ourselves down to molecular size and look at what cells do, it becomes clearer that cells somehow invented a new kind of molecular-level intentionality—a way to partly overcome the deterministic thermodynamic buffetings to which all matter is subject. This does not imply that cells create mysterious, irreducible holistic forces; in fact, we have a deep mechanistic understanding of how they work. But it is a natural way of characterizing what goes on in cells that distinguishes it from the prebiotic chemical cycles in the atmosphere, in volcanism, exposed mountain ridges and sedimentation in rivers, shores, down to the ocean floor, all of which involve an energy-dissipating, order-creating, but non-biological kind of ‘evolution’.

The chemical soups out of which life arose were already complex systems containing many different types of dynamically stable units. For example, it is thought that among other things, prebiotic soups contained isolated amino acids, the eventual constituents of biotic protein chains. Cells, however, invented a way to encode, use and reproduce information about how to cause thousands of different chemical reactions in this soup to happen. The tricky part is that the code strings, as well as all of the interpreting apparatus for them, had to be in the soup where everything was still subject to the soup's deterministic buffetings. At the outset, proto-information must have been somehow partially hidden from the degradative attack of the soup. But once cells came online, they were able to speed up many chemical reactions, prevent others, invent new ones that never used to happen at all, and above all, order, organize and compartmentalize the chemical reactions. In short, code-using cells took over forceful control of chemical phenomena in local regions of the otherwise still prebiotic soup at the energetic expense of their surroundings.

But this ‘evolution’ from prebiotic to biotic systems was not modern Darwinian evolution. Until most of the code-using system was in place, bona fide Darwinian evolution as it is usually defined—heritable variations in fitness [20]—was not possible. Darwinian ‘heritable variations in fitness’ pre-supposes a genotype/phenotype distinction and full cellular intentionality—not mere replication in a biochemist's well-supplied reaction chamber. The central problem of the origin of the coding system in cellular life is to try to come up with *pre*-Darwinian reasons for how such an intentional system might have arisen out of prebiotic situations lacking intentionality.

## 3. A second origin

In thinking about the origin of the higher-level symbol-using system in human language and culture, the situation is quite a bit more complex as human language was built upon a pre-existing cellular genetic system that was already capable of Darwinian evolution. Despite huge differences in scale, it is a remarkable fact that the fundamental rate of meaning assembly in these two very differently scaled systems is almost the same—a handful of amino acids per second, a handful of word meanings per second. Despite this similarity, language-based cultural evolution is much faster than biological evolution; lately, it has gotten so fast that biological evolution is effectively stationary by comparison (there is precious little ‘nature untouched by humans’ left). The faster speed of cultural, language-based evolution is largely attributable to the fact that cellular symbols strings (DNA and RNA) are comprehended (turned into protein meanings) but never *produced* from meanings; cells instead have to wait a long time for favourable mutations to occur. Human language, by contrast, is a more dynamic, free-for-all, two-way system in which people willy-nilly inject mental-reaction-controlling speech symbol streams into each other's brains [13]. The great difference in effective evolutionary velocity has made it difficult for transient language- and culturally-transmitted memes to be fixed in the much more slowly evolving genomes of long-lived humans. Despite their great reach and elegant variation, humans are much more genetically similar to each other than even small local populations of most other animal species.

But there is one great point of interaction between the DNA-based genetic system and the language-based human cultural system—the genetic basis of the peculiar human ability to readily learn a language. It seems seductively natural to try to come up with Darwinian evolutionary explanations for why this might have occurred, in the spirit of evolutionary psychology. By contrast, I think we may be able to make more progress by considering the *origin* of language as essentially a *pre*-evolutionary problem—that is, as the second origin of a symbol-using, and evolution-supporting system, one that partly relies on DNA-based symbols for its construction and persistence, but that is largely decoupled from biological evolution in its form and content.

In fact, human language might best be thought of as a brain operating system that allowed us to partly overcome the constraints on the Darwinian biological evolution of behaviour in much the same way that cells have partly overcome the deterministic constraints on the ‘evolution’—now in the physicist's sense of ‘the evolution of a dynamical system’—of prebiotic soups. From this analogical perspective, the origin of language may have involved the reinvention of the trick of hiding information ‘in plain view’ from the dissipative attack of the ‘soup’. But this time, the ‘soup’ consisted of Darwinian constraints on the evolution of animal behaviour. Instead of using Darwinian evolution to explain language, our goal is to see how the origin of language *circumvented* evolutionary psychology.

## 4. The ‘semantic urge’

An assumption that lies behind many language origin scenarios is something that I have called the ‘semantic urge’. This intuition grows out of the fact, just mentioned above, that the human linguistic coding system was built on top of a lower-level biological coding system that was already capable of constructing sophisticated, non-linguistic cognitive systems such as those in parrots, whales, dogs and primates. The sustained goal-directedness of animals makes it very hard to avoid the notion that human language must have somehow grown out of an insistent craving of inarticulate homininds to communicate complex meanings to each other, perhaps initially by gesture, given that vocal motor control of the kind possessed by the primates most closely related to us probably was not up to the task.

This is at heart a Baldwinian picture, where behaviour provides a selective context that drives standard Darwinian evolution [21]. As noted above, there is little tendency to fall back on intuitions like this in thinking about the origin of cellular life because cellular life had no analogous pre-existing, code-using system capable of goal-directed behaviour beneath it. For example, one influential picture about the origin of life is that proto-symbol chains emerged first without standing for anything and then only later were taken over as a code for other meaning chains that could fold up and control chemical reactions. There is no Baldwinian semantic urge of prebiotic soups to control their surroundings that drives the emergence of cellular proto-symbol chains. The picture of proto-language that we arrive at by taking this analogy seriously is somewhat peculiar; but it fits much better with what we know about the evolution of vocal behaviour in other animals (see also [9,13,14,22,23]). Before doing that, let's first review some of the evidence for the origin of symbols and proto-symbols at the cellular level.

## 5. The ‘RNA world’ without a semantic urge

The original idea of an ‘RNA world’ [24] was independently proposed by Woese [25], Crick [26] and Orgel [27] as a predecessor of modern DNA/RNA/protein-based life. It gained major support from the unexpected demonstration in the early 1980s that RNA could act as a bona fide, enzyme-like catalyst (an RNA segment in single-celled *Tetrahymena* was discovered to fold up and catalyse RNA-splicing). The foundational role of RNA is obvious from observing its current position in cells (recent review: [28]).

Unlike other components of the cell, RNA can act *either* as a one-dimensional symbol string (messenger RNA) *or* as a three-dimensional self-folding controller of chemical reactions (structural RNAs, based on the protein-like ability of RNA to form precisely shaped surface cavities with high specificity for particular substrates). This crucial Janus-like ability is used sparingly; modern cells mostly employ proteins, not RNAs, to control and catalyse the thousands of chemical reactions they run. The instances where RNA is used as a protein-like structure, however, stand at the very centre of code-use in cells, including (i) RNA editing (in the nucleolus, which generates ribosomes, and in the spliceosome, which edits messenger RNA), (ii) recognizing words (codons) in code-like messenger RNA strings (by transfer RNA) and, most importantly, (iii) using the sequence of recognized words in messenger RNA code strings to assemble individual amino acid meaning units into functional protein chains (by ribosomal RNA).

The idea that the dual roles of RNA as code and catalyst might have bootstrapped life gained support over the years as additional catalytic RNAs were discovered, and especially after large-scale structural RNAs (e.g. ribosomes) were finally crystallized showing that it was the RNA itself, not the associated proteins, that directly catalysed the attachment of each coded-for amino acid ‘meaning unit’ onto the growing protein chain [29–31].

## 6. The origin of proto-information: before the RNA world

Despite the intuitive plausibility of the ‘RNA world’, however, it has proved to difficult to find plausible prebiotic synthesis pathways for nucleotides, the subunits of RNA [32]. This stands in sharp contrast to the easy prebiotic availability of amino acids [33,34], the eventual molecular units of meaning. Many origin-of-life researchers were led to search for chain-like prebiotic precursors of RNA itself based on more easily obtainable subunits than nucleotides [35]. A key feature of this search—so obvious to those within the field of prebiotic synthesis that it is rarely explicitly stated—is to find reasons *other* than the ability to code for amino acid meanings as to why a pre-RNA-like molecule might have come into existence.

Once pre-RNA or RNA or existed, its dual role as a catalyst (protein-like) and a code-chain (DNA-like) could then be discovered, leading to something like an RNA world [36–38], and then finally to modern DNA/RNA/protein life, where code-only DNA and meaning-only proteins occupy opposite ends of the spectrum from symbol to meaning.

## 7. Problems with the semantic urge in the prelinguistic world

In turning back to language, many language origins scenarios start with a repertoire of already meaningful vocalizations like those used by many different animal species [39] and then attempt to come up with a reason—typically, the semantic urge—for why they might have multiplied [40,41]. Several people [42,43] have pointed out that there is a major problem with this standard scenario. Animal calls—such as the well-studied set of vervet monkey alarm calls [44], but also calls in many other animal species—are laden with emotional meaning. The usual explanation for this is that alarm calls often are emitted in life and death situations, which generates strong selection pressure to maintain call reliability; this reliability has been ensured across many diverse species by tightly neurally tying calls to the emotional state of both the sender and the receiver. This tight linkage, however, presents a problem for the scenario of calls as a starting point for proto-language; the number of different emotional states is rather small, and emotional states do not follow each other in a quick, regular succession like words must do (see below, on the need for speed).

The origin of language required the development of a large inventory of sound combinations in order to code for thousands of meanings that are made more specific by assembling them into strings at the rapid rate of several words per second. Individual word meanings—especially for high-frequency polysemous words central to every language, such as ‘over’, ‘put‘, ‘give’, ‘line’, ‘big’ or ‘hand’—are freed from emotion when compared to animal calls. Certainly, some single words such as epithets can be intrinsically emotive, but these are in a small minority. Perhaps, the difficulty of imagining a path from a handful of emotive calls to the 5000-word core of mostly emotionally neutral words in human language stems from the fact that the two are phylogenetically unrelated. The analogy with the RNA world and the pre-RNA world suggests that perhaps we should instead try to find a way by which a large pool of pre-RNA-like pre-words might have been generated—units that are like words in many sensory and motor respects, but that *do not* yet stand for anything.

In the cellular situation, the bonds between any pair of RNA nucleotides in a chain are approximately equally stable. In Sereno [13], I called this arbitrariness_2_ (to distinguish it from classical pairwise symbol–object Saussurean arbitrariness_1_). Arbitrariness_2_ can be thought of as a trick to prevent the prebiotic soup from recognizing and thus selectively destroying the incipient information ‘hiding’ there in the form of different sequences. For pre-language, we may have needed a similar trick to prevent individual units or small groups of them in incipient speech symbol chains from (initially) being recognized as meaningful. Iconicity—but also any accidental arbitrary_1_ (Saussurean) attachment of the vocal pre-symbol groups to emotional meaning—may have actually been a Darwinian evolutionary *impediment* to the development of a large enough catalogue of vocal pre-symbol segments. As in the molecular case, proto-information at the linguistic level may initially have been hidden from the ‘soup’ of animal behaviour ‘in plain sight’ to allow it to accrete, and to avoid having it attacked and destroyed by having it elicit particular behaviours in other animals, which would then drag everything back into the simplified sound features and semantics, and automatic emotional binding of isolated alarm calls and affiliative calls.

## 8. Information versus proto-information

In Shannon–Weaver information theory, the more random and unpredictable a signal on a channel is (by analogy with chemical entropy), the more information it can potentially convey [45]. An image with large patches of white and black transmitted pixel by pixel is a very non-random signal. If this image is first compressed—e.g. by sending the number of white or black pixels in an all-white or all-black run rather than each pixel separately—the signal would be shortened while containing the same amount of information, but it would also begin to look more like unpredictable random noise. Information theory assumes that the channel—but also the machinery at either end that can send and understand the message—are both already there.

In thinking about origins, we have instead to think about how the channel, interpreters and most importantly the one-dimensional strings themselves came into being in the first place. There is an endless supply of ‘uselessly random’ things in the world that are not information or even proto-information because there is no way to access the strings in them, or because they do not even form one-dimensional strings at all (like disconnected gas molecules). In contrast to usual language origin scenarios that start with single meaningful Saussurean sign–object pairs, the perspective here is to look instead for plausible sources of *proto*-information *strings*—currently unused, meaning nothing, but *potentially* usable. Good quality proto-information should be random-appearing, like efficient Shannon information, which requires the ability to attach symbol segments into one-dimensional strings in roughly random orders. But just as critically, there needs to be a pre-existing productive mechanism for generating and processing these one-dimensional strings in plain view, so they could eventually be integrated into a meaning delivery system, but with their content initially hidden because of their apparent randomness.

Strong one-dimensional chains (of nucleotides, amino acids and saccharides) are what principally physically distinguish the current biotic molecular world from the prebiotic world of rocks and minerals. These chains are held together by strong covalent bonds in aqueous solution. Rocks and minerals are certainly stronger than biomolecules when dry; but once dissolved in water (where all the action is), the chains and lattices in rocks and minerals all break down into small pieces. Human language is distinguished from animal communication in an analogous way—the towering sequence complexity of the long one-dimensional chains in human language set it far apart from meaning-carrying signal systems in every other animal. The analogy with the RNA world suggests that we look for sources of meaningless proto-information chains rather than the more traditional and intuitive approach of trying to find ways of making sentence-like chains out of already bound together but isolated symbol-referent pairs. Evidence from the evolution of animal vocal behaviour provides intriguing sources for such meaningless chains.

## 9. The example of birdsong

The possible relation between birdsong and speech was noted early on. Darwin—who in *Origin of Species* [46] often discussed the relation between biological and linguistic evolution (interestingly, to argue that biological evolution might be like language evolution, not *vice versa*!)—turned briefly, in the *Descent of Man* [47], to language origins. Darwin was especially fond of scenarios in which a structure had initially evolved for one purpose only to become a ‘preadaptation’ for another. Breaking with the common sense view that language arose from a gestural substrate, he suggested that language developed out of a form of ‘rudimentary song’, a kind of purely prosodic pre-language that conveyed emotions and other broad, unitary meanings in much the same way that pitch modulation and emphasis are used in modern speech. Darwin mentions the flashy hooting vocalizations of gibbons, which are generated during territorial and courtship displays, as something like what he had in mind, but pointed out that birdsong provided ‘in several respects the nearest analogy to language’ ([47], p. 55), citing the work of Daines Barrington a century before [48] on the extended learning period for birdsong, the initial ‘babbling’ stage and the development of birdsong dialects.

Modern research on birdsong has provided a neurobiological foundation for these earlier hunches, but has also revealed a system that looks a good deal more like human-style, ‘left-hemisphere’ speech than like the call systems of other animals (including gibbons) [10,49–51], but also the call systems of songbirds themselves, who have retained their limited set of emotional calls alongside song. There is a powerful perennial tendency outside fields explicitly focused on evolutionary processes to think of evolution in terms of a ‘Great Chain of Being’ and to ignore the mosaic nature of evolution. Thus, birdsong has often been dismissed as a model of human language for the reason that monkeys seem much smarter than some birds, or that monkey calls seem to have more semantic content than birdsong. In fact, the importance of birdsong in the present context (only dimly glimpsed by Darwin) is exactly the fact that a set of language-like features have evolved *in the absence of* a semantic function.

Birdsong requires a significant learning period, during the early parts of which the young bird is silent. If a bird is not exposed to a tutor song within a certain early critical period, it will produce only a crude version of its species’ song. Normally exposed young birds initially produce sounds called subsong that resemble the progression of types of babbling in baby humans—initially a broad range of sounds are produced, followed by an unorganized recombination of species-specific song fragments, and then finally, adult song. Within a species, there are regional dialects that are learned from a bird's regional peers; artificial rearing experiments show that birds learn the dialect of their tutors, regardless of their genetic background. Adult song repertoires can be considerable; some wrens produce hundreds of distinct songs, each containing 5–20 ‘syllables’, while mockingbirds produce virtually endless sequences of different syllables in variable orders. Good singers may have a thousand or more distinct ‘syllables’ (a ‘syllable’ consists of a particular figure sometimes repeated once or twice; in this respect, it is unlike a phonetic syllable, which consists of one or more consonants and a vowel). If a songbird is deafened before learning to sing, it will fail to produce song-like sounds as an adult. By contrast, non-song birds and many other animals including non-human primates (e.g. gibbons) that do not learn complex serial vocal patterns from their peers, still come to produce their species-specific sound repertoire when deafened at birth [52]. In many respects, it might be more accurate to call it ‘birdspeech’, because birdsong differs from human singing and musical performance in many ways; for example, birdsong lacks a regular metre, musical tonality and harmony (though see European starlings on ‘harmony’ in §13).

The parallel evolution of fine-grained vocal control in singing birds affords a crucial comparative perspective on the anatomical and neural constraints on auditory–motor learning and performance. Birdsong is initiated in a structure called the syrinx, which is evolutionarily related to (and controlled by the same nerve as) the tongue. It is generated primarily by directly controlling the fundamental frequency produced by the syrinx. Human speech sounds, by contrast, are generated by filtering and modulating the higher harmonics of the fundamental frequency of the vocal cords in the larynx (by controlling the position of the tongue in the pharyngeal and oral cavities), making the higher frequency parts of speech sounds independent of fundamental frequency (voice pitch). Nonetheless, in many respects, birdsong is much more like human speech than are the vocalizations of other animals, some of which can even modulate laryngeal harmonics (for example, monkeys [53], male deer [54] and seals [55]) in a human speech-like fashion.

There are intriguing clues about the evolution of fine vocal control from neuroanatomy of the avian song system. For example, motor output neurons in the forebrain (in the robust nucleus of the arcopallium, RA) of songbirds have gained direct access to motoneurons controlling the syrinx vocalization musculature. Projections from RA bypass the brainstem pattern generator circuitry for calls through which all forebrain outputs must pass in non-song birds like ducks [56,57] but also in squirrel monkeys [58,59] and macaque monkeys [60]. There is a striking parallel here to the evolution of fine finger control in primates (but also finger control in raccoons, as a yet another reminder that evolution is a bush, not a linear Great Chain of Being), where motor cortex neurons have also come to contact finger motoneurons directly, bypassing pattern generators for coordinated limb movement situated in the spinal cord; hand motor cortex in cats, by contrast, contacts primarily the spinal pattern generators, which then have the only private access to motoneurons. The more direct access afforded to the forebrain in the case of the songbird syrinx and the primate and raccoon hand presumably underlies more complex, differentiated, learned control of these effectors. Note that this means that the relevant forebrain motor output areas have essentially come to assume a *lower* level in the motor control hierarchy, allowing the development of other forebrain pattern-generating centres that can operate in parallel with and independent of the brainstem and spinal pattern-generating circuitry that is still needed for locomotion in the case of the hand, and non-song vocalization in the case of the syrinx.

## 10. Speech-like birdsong carries *less* meaning than vocal call systems do

The most striking characteristic of birdsong, however, in light of its prodigious complexity, is its essential lack of semantic content. Individual syllables or song fragments do not seem to have any specific meaning outside of being part of a particular song; and particular songs do not seem to convey specific content. Nor do birds appear to produce anything like ‘words’ by recombining their ‘syllables’ in order to signify concepts. Despite having motor, vocalization and auditory equipment ideally suited to support the re-combinable speech-symbol half of a language-like meaning-conveying system, birdsong seems to communicate only very general meanings. Songs serve to mark territories, identify the singer's species, attract mates and cause ovulation, often all at once. The messages communicated by birdsong are, in fact, *less* content-filled than the messages communicated by, for example, vervet monkey calls—which have been shown to signify rather elaborate distinctions among predators and conspecifics [44], despite the fact that these unlearned, unitary calls are drastically simpler than birdsong. This difference in referential content is particularly obvious when we consider the ‘meaning’ of a handful of syllables of a songbird's song; though emotion is keenly involved in motivating the bird to begin singing, the identity and order of syllables carry no additional specific emotional baggage.

Attempts to find particular ordering patterns in bird song are a topic of hot debate, particularly with respect to what level of complexity of song grammars birds are able to recognize after training [61]. Without visiting that particular debate, in the case of natural song sequences, researchers have shown that song order is not random, but can be modelled with hidden Markov models [62]. However, in support of the present line of argument on the meaninglessness of sequences in birdsong, there is little evidence that the different naturally occurring syllable and song orders themselves signify different things, at least from observational studies of the behaviours of singer or listener birds.

## 11. Sexual selection and birdsong

One plausible theory about birdsong is that it was a product of runaway sexual selection—like the male peacock tail or outsize antlers in male deer, or huge inconvenient-looking sexual swellings in female baboons and female chimpanzees. Elaborate singing abilities seem to have been preferred by mates, despite making little direct contribution to fitness beyond the fact that they were preferred. Sexual selection stands in contrast to natural selection, which rewards improved function like a stronger beak or more efficient wings. Certainly, a complex song can serve as a sign of a mate fitter in other non-song respects. It is a little more difficult to explain the maintenance of extreme examples this way, especially when sexually selected features run the risk of impeding other functions (huge antlers) or attracting predators (elaborate vocal displays). Zahavi [63] has suggested that these handicaps have evolved to serve as an honest signal; the feature advertises that the animal was fit enough to overcome the handicap. Though empirical support for the handicap model from animal studies has been mixed, there is little doubt that sexual selection in general can drive evolution in a different direction from natural (functional) selection.

Sexual selection is not confined to female choice affecting male characters (see primate sexual swellings above). And both the male and female sing in some songbird species. Bay wren male–female monogamous pairs, for example, execute precisely coordinated ‘duets’ where the pair trade singing back and forth several times a second, creating what sounds to an untrained ear like the song of a single bird (see example in [23]). In these birds, the song control nuclei are large and hormone-sensitive in females as well as males [64]. The generally accepted explanation for this behaviour is that the attractiveness of the male's song to listening females is reduced when a duetting female is heard intimately trading back and forth with that male.

Several whale species have independently evolved a vocal learning system that resembles birdsong in a large number of respects and provides a key additional example of how a speech-like vocal learning system can evolve without a ‘semantic urge’ [65]. Humpback whales learn to precisely reproduce long sequences of sounds and culturally transmit them to animals that are genetically unrelated. The main difference is that whale songs are lower in pitch, and individual songs unfold over a minute instead of several seconds. The underwater acoustic environment of the ocean is quite reverberant, due to the faster and more efficient propagation of sound in water and as a result of reflections from the air–water boundary. This may be one reason for whales' more leisurely tempi. As with birdsong, whale song has social and sexual functions, and precise, lengthy sequence perception and generation.

## 12. The need for speed

One idea implicitly introduced above was the notion that meaning assembly in language might demand a certain minimum speed, like flying. At first, it might seem that there is no ‘minimum speed’ for language as strongly motivated humans (e.g. Stephen Hawking) are capable of comprehending and producing language at very slow rates (e.g. one word per minute). However, in the context of the origin of language, for a proto-meaning-assembly process to be useful in a social context among proto-linguistic animals, it is less clear that such extremely leisurely rates would be practical; the chance of non-linguistic interactions and events disturbing the meaning assembly process increases as the interword time goes up. A second motivation for speed is that if language meaning assembly piggy-backed on non-linguistic visual scene assembly (see below), the word-meanings-per-second rate might initially have had to more closely match the typical rate of uptake of sequential glances used by the visual system (several new fixations per second).

These two considerations may provide additional independent motivations for why elaborate, rapid, but non-meaningful perceptual and motor *string sequencing* might have had to evolve first in order to boot language. These arguments are relevant not only for vocal signalling but also for visuomanual signalling. It is important not to forget that a small set of meaningful alarm calls and meaningful gestures have independently evolved in a very large number of different animal species for a half a billion years. The evolutionary advantage to a species of being able to communicate more complex meanings would have been (and remains) great; that is, there must have been countless opportunities to extend unitary pre-existing meaningful calls or gestures, but none were able to be taken.

## 13. A birdsong-like ‘RNA world’ for pre-language

With the context provided above, we can see our way to a surprising extension of Darwin's language-origins theory. On the evidence of the avian case, it seems possible that early hominids might have initially evolved an elaborate system of essentially phonetic vocalizations—a kind of ‘song talk’ with no attached semantics—as a result of sexual selection. In this view, a number of the specializations for speech-related auditory–vocal control evolved for entirely non-semantic reasons. Perhaps early hominid pairs ‘duetted’ like bay wrens for several million years before reference was invented. At first, it might seem unreasonable to imagine almost fully developed human speech without meaning; but the surprising variety of birdsong—and the parallel whale example—suggest that the evolution of the ability to generate elaborate but meaningless vocal sequences is a reasonably common, evolutionarily stable strategy. Turning standard Baldwinian language origins scenarios on their heads, the pre-adapted ‘symbols’-without-meaning system might have only been taken over for use as a semantic vehicle at the very last moment. The scenario of talking before referential speech is odd, but no odder than birdsong and whale song themselves.

This scenario contrasts with Fitch's idea that laryngeal descent in hominids (which occurs early in postnatal development) might have been used as a strategy to indicate large size in males, by analogy with the realtime laryngeal descent that occurs during calls made by rutting male deer [54]. A functionally similar kind of call, though using resonant air sacs instead of laryngeal descent, is well known in gibbons and orangutans, and it closely resembles emotional-meaning-laden signals in standard animal call systems. Unlike birdsong, these primate calls develop even in deafened animals, indicating that learning is not required.

The birdsong model suggests instead that there was runaway selection for complex *sequences* of essentially meaningless segments—each individually untied from particular emotions—as opposed to selection for a large-sounding roar or a deep voice. This is not to detract from throaty roars, which are a common theme in male animal vocalizations; but elaborate sequences can be just as attractive as deep throatiness. Different bird lineages have explored many intriguing ways of attractively increasing song complexity. The superb lyrebird (‘superb’ is part of the common name) accurately imitates sounds in its forest environment, including the sounds of other birds and other animals, and poignantly, the sound of chain saws, attracting mates by means of a large and impressively detailed repertoire [66]. European starling males can independently manipulate the two halves of their syrinx during singing to create a ‘one man band’ effect with a lower frequency ‘bass line’ overlaid by asynchronous higher pitched notes. Experiments with playback show that the more complex songs sung by older, more experienced starling males are more effective at inducing ovulation [67]. Finally, although male plus female singing like the wren example discussed above is less common than male-only singing, it has nevertheless evolved in more than one lineage (e.g. female superb lyrebirds are quite competent imitators), overcoming objections (e.g. [68], p. 96) that sexual selection cannot explain female speech.

## 14. Analogues of structural and catalytic RNA in the auditory system

As mentioned above, RNA molecules serve both as a code (messenger RNA), but also critically as non-code-like, self-folding word-recognition devices (transfer RNA) and catalytic meaning chain-assembly devices (ribosomal RNA). By analogy, the internal representations of speech sound sequences that a primate neurobiologist would expect to find in the human superior lateral temporal cortex may have acquired other functions besides merely serving as internal copies of the speech stream. Perhaps there was a leisurely ‘RNA world’-like stage as sexual selection was shaping vocal learning, where RNA-like speech sound representations in the auditory temporal lobe interacted with each other and gradually increased in complexity *without* being attached to meanings (e.g. visual meanings).

Then at a later point, the ‘catalytic’ abilities of uninterpreted speech streams were suddenly exposed in the service of attaching visual meaning representations into chains in a manner similar to the ribosomal catalysis of amino acid chains. Non-symbolic functions for internal representations of uninterpreted speech streams is a strange idea that would require more evidence than we currently have for it to be taken seriously; however, the central role of structural/catalytic RNA in protein synthesis (as opposed to being a mere code-like messenger) is arguably just as strange—and it took many years before that idea was finally fully accepted.

Nothing has been said (1) about how the internal representations of speech sounds got connected to visual meanings, or (2) about the dynamics of how concatenated visual meaning patterns interact in the complex ‘mental metabolism’ that must be present in linguistically competent human brains. But perhaps that second bit did not have to be invented out of whole cloth. Instead, it could have piggy-backed on an already existing system for assembling visual inputs arriving from early visual areas during the process of *visual scene comprehension*; the higher-level visual system was already an expert in the rapid serial assembly of successive glances. The implication is that the trick of language was not to have invented the basic meaningful units—nor even the rules for how concatenated chains of visual units self-assemble—but merely to have found a symbol-string-directed way of making standardized connections between them [3,11–13,15].

## 15. Language as code-directed scene comprehension

Vision is very important to primates; in fact, almost half of the cortex in primates consists of areas that are specialized for visual processing. Primate auditory and somatosensory areas each cover about one-quarter as much cortical surface area as visual areas do [69,70]. Together with the fact that virtually all anthropoid primates (monkeys, apes and humans) are diurnal, it makes sense for a substantial core of concrete word meanings concerning objects, properties, actions, manners and paths to be represented in the visual system. The idea that visual representations may be important in the semantics of natural language [3] has been around for a long time (in linguistics; see [71–74]). Common to those approaches is the notion that concrete visual meanings have been extended via analogical processes to deal with more abstract objects and relations. An unremarkable sentence like ‘I think I got my idea across to him’ uses unmarked ‘get’, ‘across’ and ‘him’ as if the abstract concept ‘idea’ were a physical object being transported across a physical bridge from me to him. The present proposal goes further in suggesting a particularly direct moment-to-moment relationship between the mechanisms of scene and discourse comprehension.

Language—especially when transcribed to text—quite obviously has a fundamentally serial nature. At first, vision might seem to be less serial. This is in large part because we cannot as easily and compactly ‘write’ vision as we can language. But from the point of view of primary visual cortex (or the view of a filmmaker), the integration of successive glances in the comprehension of a visual scene requires a kind of serial assembly operation similar in a number of respects to the serial integration of word meanings in discourse comprehension. Primates make long series of fixations at the rate of several new views per second during scene comprehension. Each fixation brings the high-receptor-density fovea of the retina to a new part of the visual scene and generates new activity in V1 dominated by objects at fixation, which largely displaces the activity there caused by the previous fixation. Higher visual areas with less precise retinotopy somehow integrate information from these disconnected and distorted (centre-magnified) activity sequences across time (e.g. [75]) to generate an internal representation of the location, identity and relations of the relevant objects in the current scene that serves as a basis for moment-to-moment action. There are a number of aspects of this initially strictly serial process that are strongly reminiscent of serial meaning integration in language comprehension.

For example, isolated glances taken out of context are as underspecified and polysemous as a single word taken out of discourse context. The context-free information available from an isolated 250 ms glance at a common object—e.g. a leaf on a nearby branch—could mean a lot of different things in the context of preceding and following glances—e.g. something to walk on (the branch), something to eat, something to duck under, something to grab onto to correct balance, something to brush aside, something to shake or something completely irrelevant. The specific visual meaning in a single glance is only sharpened and fully developed after considering the context of the full train of glances (and motor state) that came before and after it. This is similar to the integration of linguistic meaning from word strings. An isolated 250 ms experience of a high-frequency word like ‘line’ taken out of context is as polysemous as the glance at a leaf on a branch; it could be about ‘line up those objects’, or ‘line up a supplier’, or a ‘line of kings’ or a ‘line of thought’, ‘in line with what I'm thinking’, or ‘don't cross that line’, or prosaically, ‘a clothes line’. Though the meanings conjured up here are often listed in a dictionary under ‘line’, most of the work may instead get done by interaction with context, as in the case of the leafy branch; a truly isolated experience of the unique semantic content of ‘line’ (with anaesthesia before and after) is probably much more minimal than what is found in the dictionary. The impetus for thinking this way came from considering the truly *un*remarkable chemical properties of isolated amino acids—the word meanings at the molecular level—compared to their incredible, multifarious catalytic specificity upon their mere concatenation into self-folding amino acid chains.

Second, there is a physical divide in the visual system between motion processing—in the middle temporal area (MT), the medial superior temporal area dorsal division (MSTd), the lateral intraparietal area (LIP), the ventral intraparietal area (VIP)—and object property processing—shape, colour and object identity in the fourth visual area (V4) and inferotemporal areas. Though the properties of neurons in the two pathways are somewhat less distinct than once thought (e.g. [76]), and recent comprehensive analysis of connections has turned up a large number of additional (though numerically sparse) connections [70], this basic division remains the first principal component of organization in the visual system. In language, there is a similar primary divide between noun/adjective and verb/manner/path in phrasal syntax.

Third, information from temporally distant glances fixating roughly the same thing must be tied together, as with linguistic anaphora. For example, an initial glance at a particular monkey's face might be followed by glances at a branch, the ground, a different monkey, a cloud (looking for an eagle) or a flower, before the same monkey is finally re-fixated. At that moment, the particular monkey must be re-identified (e.g. the fixation might have fallen on the target monkey's shoulder rather than the face, or mostly in the opposite hemifield) and then information from the previous fixation must be integrated with information from the current fixation; for example, that particular monkey could be looking more aggressive than he was at last glance, or now he has turned away, or now he is eating a small plant root, or now he is in the same position and mood as he was in the previous glance.

In both signed and spoken language, there are a variety of long distance anaphoric dependencies and interactions established as a serial discourse is played out. These range from pronouns ‘I told John about the job; *he* agreed to do *it*’, to pronominals, to more complex pointers in realistic multisentence discourses. For example, I could metonymically refer to the content of the entire previous paragraph with ‘that crazy “visual anaphora” idea’.

None of this implies that scene representations (or their presumed linguistic fellows) need look anything like pictures; the patterns in question are very likely distributed across many areas, a number of which show little retinotopy.

One main difference between scene and discourse comprehension is, of course, that scene comprehension is tied closely to the current scene. Discourse comprehension could be thought of as a kind of fictive visual scene comprehension directed, in the case of spoken language comprehension, by sequences of phoneme representations in secondary auditory cortex. The obvious advantage of linguistic discourse comprehension is that we are no longer tied to the current scene. However, once the appropriate visual word meaning patterns have been called up and bound together, the nature and interactions of the composite pattern might be conditioned mainly by the prelinguistic rules of interaction of scene representations in primate visual areas networks. In this sense, a part of what has been called linguistic syntax and semantics might not be modular with respect to the neuroscience of vision.

There is in fact substantial evidence that visual areas in humans are involved in specifically linguistic functions. There is a kind of aphasia that is self-contradictorily called ‘transcortical sensory’ aphasia (i.e. ‘across-from-the-language-cortex’ aphasia!) that is generated by a lesion in left human inferotemporal cortex [77]. Many of these lesions are so posterior and ventral that they are associated with overt visual field defects. Transcortical sensory aphasics have poor, Wernicke's-like comprehension, yet paradoxically (at least in the context of traditional models of language comprehension) can repeat words effortlessly. Far from being ‘across from the language cortex’, the visual areas in inferotemporal cortex damaged in these patients may be one primary site of semantic processing in sighted humans. More recent cortical stimulation studies in intact (but epileptic) brains have demonstrated language arrest from inferotemporal cortex stimulation ([78]; see also [79]) and have also shown that middle temporal gyrus stimulation (between auditory superior temporal cortex and visual inferior temporal cortex) can create transcortical sensory aphasia-like behaviour (preserved repetition with poor comprehension) but *without* impaired naming, pointing to the middle temporal gyrus region as a key bridge point between input phonology and more inferiorly located visual semantics [80].

Transcortical sensory aphasics recover more quickly than patients with more superior lesions, but this may only be an indication that the functions performed by visual cortex in language comprehension are less lateralized than those performed by auditory cortex and face motor cortex. This is consistent with what we know about primate temporal visual areas; it has long been known that permanent deficits in visual pattern recognition in monkeys require *bilateral* inferotemporal cortex lesions [81]. There is no need to assume that all the cortical areas involved in language comprehension are equally lateralized.

Psycholinguistic experiments using pictures inserted into sentences and priming between words and pictures [82,83] suggest that it is surprisingly easy for visually represented concepts to be integrated into ongoing linguistic discourse comprehension or for words to directly activate visual perceptual representations. This may be another indicator of the closeness of visual category representations to linguistic meanings. Certainly, it is easier to make little pictures or movies to represent a large range of word meanings than it is to make non-linguistic sounds (how to purely auditorily represent ‘over’ or ‘give’?).

It is certainly not necessary to site all meaning in the visual system. There is a rich world of non-symbolic auditory and somatosensory experience. And a similar serial scene assembly process to that described above for vision, but for sequences of sounds or touches, must be done in those modalities, too. And there are many other animals for whom vision is not the primary distal sense, such as echolocating bats or electric fish (many of which are nearly blind); bats and electric fish must rely primarily on serial assembly of auditory and electrosensory ‘glances’; for a bat, it would probably be easier to comprehend an auditory ‘picture’ of ‘over’—a small object moving over a larger one—than it would be for it to comprehend a visual one.

## 16. Iconicity in sign and speech

At long last, we are positioned to consider iconicity and the semantic urge in sign language.^1^ Individual signs often contain handshapes, places of articulation or movements that relatively straightforwardly refer to one or more parts of an object, action, property or manner. Iconicity continues to affect online lexical processing of signs in adults [84,85]. And iconicity in sign language extends into sign language syntax, in the form of spatial pronominal and deictic reference, and as classifiers that modify subjects and verbs. Iconic signs can vary across languages (e.g. ‘tree’ outlined with two fingers versus represented as a trunk and branches with a raised forearm). The referent may be difficult for a sign language-naive hearing observer to guess; Klima & Bellugi [86] classified the iconicity of signs on a scale ranging from transparent to obscure, based on how evident the mappings were to naive observers. But there is little doubt that iconicity is more prevalent in signed languages than it is in spoken languages. By Klima and Bellugi's measure of transparency, the most strongly iconic speech examples (outside of a class of onamatopoetic speech sounds and ideophones) would probably rate as ‘obscure’—for example, the high vowel for ‘me’ or ‘here’ may both indicate closeness to the speaker, while a lower vowel for ‘you’ or ‘there’ may indicate greater distance.

The primary reason for this visual/auditory difference in the prevalence of iconicity in sound and sign is simply that the visual system dominates the primate neocortex, making vision the modality with the greatest iconic potential; it has the most diverse set of representational machinery for characterizing objects and features, and actions, paths and manners. But vision did not always win. We have already mentioned bats and electric fish. But consider catfish, which remarkably use taste as a distal sense; the catfish brain has detailed and repeated maps of taste buds [87], which are distributed across not only its barbels and face, but cover its body all the way down to the tail, and higher-level areas in the catfish brain must do serial assembly of gustatory glances.

Another reason that visual iconicity is easier than auditory iconicity is the intrinsic dimensionality of the respective receptor surfaces and cortical maps. The visual receptor surface is two-dimensional (eccentricity and polar angle) while the auditory receptor surface is one-dimensional (frequency). Spatial coordinates and relations among actors and objects in scenes take a lot more work to construct using activity spread across auditory bandpass filters than they do from the more camera-like retinal movies relayed to V1.

From the view of this paper, a reason why language probably had a vocal origin is precisely that the more natural visual iconicity of gestures might have been an impediment to building up a large enough catalogue of meaningless pre-symbolic gesture strings in evolutionary time.

But once the neural structures responsible for the control of hierarchically structured vocal motor patterns were fixed in the human brain by Darwinian evolution, iconicity would no longer serve an impediment and could instead turn into a convenient aid to sign language learning [88]. That it is not as readily accessible in the auditory modality is auditory's loss.

Finally, the idea of language in sighted humans as primarily code-directed *visual* scene comprehension introduced above provides a rationale for why auditory symbols might initially have been preferred as opposed to visuomanual symbols; given a vision-dominant diurnal animal, there would be more overlap and potential interference between symbol strings and meaning strings in the second case.

## 17. Conclusion

Human auditory–vocal language was unlikely to have arisen by the multiplication and concatenation of the small set of hard-wired, meaningful, vocal alarm calls that have evolved in a large number of different animal species. Evolutionary constraints keep calls tightly bound to a small number of emotional states as honest life-and-death signals, which prevents them from being been converted into the low-cost strings of thousands of phonemes used to communicate linguistic semantics.

Rather, we turned to plausible Darwinian mechanisms—in particular, sexual selection—to explain the evolution of the neural control of complex learned vocalization attractive to mates for its complexity and variation, but signifying nothing specific. Birdsong and whale song are two parallel examples of how complex neural machinery for auditory–vocal sequence learning evolved by sexual selection *without* a semantic function. Attaching meaning to pre-symbols before they were numerous enough, or before they were capable of being put together fluently into long enough chains, risks a collapse back to a set of isolated meaningful calls. Instead, a large inventory of pre-symbols needed to be initially *hidden* from meaning, but still ‘in plain sight’, as it were. It was probably easier to hide proto-information in auditory–vocal sequences than in visual-manual gesture sequences because of greater natural iconicity associated with visual–manual gestures. This was the first pre-adaptation for language and may have taken several million years to fully develop.

The second pre-adaptation for language was a system for assembling visual scene representations from long chains of successive glances for the purpose of directing ongoing situated behaviour. This function was initially strongly tied to content of the current scene, specifically a visual scene, because vision is the primary distal sense in primates.

The origin of language may have been the result of the fortuitous coming together of these two initially independent Darwinian pre-adaptations. The result was the formation of a second, higher-level symbol-using system that has essentially detached itself from the constraints on the evolutionary psychology of vocal control that are built into the underlying Darwinian DNA-and-protein symbol-using system. The new system was also super-charged relative to the cellular-level symbol system because of the added facility for symbol-string production that is not found in the comprehension-only cellular system.

There remains the question of what might have accounted for the coming together of these two systems. After a long paper spent criticizing the idea of the semantic urge, perhaps the semantic urge is just what was needed here! The main argument above was not that the urge to mean did not exist, or was irrelevant, but rather that its typical stable evolutionary end state, discovered by countless lineages of animal species, was a system of isolated meaningful alarm and affiliative calls. Perhaps, the same urge to mean—when faced with the unique context of (i) an elaborate primate visual system and (ii) sexually selected receptive and productive facilities for vocal learning of long random-appearing strings—resulted in a new and revolutionary outcome of human language. This explanation is certainly too simple-minded; but perhaps it gives us a fresh jumping-off point.

Looking at language today, we see a multi-modal, multi-channel phenomenon that allows humans to control each other's minds in a much more specific and invasive way than any other animal can, by injecting potent linguistic symbol strings into each other's brains. Modern language has lost its fear of iconicity, both in development and adulthood. But maybe none of those features was there until the very end. A long-standing problem in the origin of human language is its sudden appearance. Modern-appearing cultural artefacts only appear on the scene in the Late Pleistocene; yet it must have taken a much longer time for the anatomical and neural structures that control human vocal language production to have evolved from their primitive basal mammalian condition seen in all other anthropoid primates. The birdsong/‘RNA-world’ picture described above provides one way out of this problem. Perhaps non-semantic, birdsong-like human speech was around for a very long time, perhaps even dating back to early *Homo* species. This could have set the stage for the emergence of a linguistic ‘RNA-world’, where the word recognition and chain assembly properties of still-meaningless speech–sound representations could be leisurely discovered, and then only grafted onto a pre-existing productive visual meaning construction system at the very end. Kendon [68] recently commented that language is ‘a poly-modalic activity today, [and] so it must have been in its beginnings’. I would agree—if ‘beginnings’ is taken to mean ‘the very last minute’.

## Funding statement

This study was supported by the Wellcome Trust, NIH R01 MH 081990, and a Royal Society Wolfson Research Merit Award.

## Endnote

1 Curiously enough, the present analogy between cellular and linguistic code-using systems was first developed while preparing a talk on iconicity in American Sign Language for David MacNeill's 1980 class on language and gesture.

## References

[1] Harnad SR Steklis HD Lancaster J (eds) 1976 Origins and evolution of language and speech. Ann. NY Acad. Sci. 280, 1–194. See http://onlinelibrary.wiley.com/doi/10.1111/nyas.1976.280.issue-1/issuetoc. 10.1111/j.1749-6632.1976.tb25508.x 827953

[2] Donald M 1991 Origins of the modern mind. Cambridge, MA: Harvard University Press.

[3] Sereno MI 1991 Language and the primate brain. In Proc., Thirteenth Annual Conf. of the Cognitive Science Society, Chicago, IL, 7–10 August 1991, pp. 79–84. Hillsdale, NJ: Lawrence Erlbaum Associates See http://www.cogsci.ucsd.edu/~sereno/papers/BrainLang91.pdf.

[4] Deacon TW 1997 The symbolic species. New York, NY: Norton.

[5] Jablonski NG Aiello LC (eds) 1998 The origin and diversification of language. San Francisco, CA: University of California Press.

[6] King BJ (ed.) 1999 The origins of language: what nonhuman primates can tell us. Santa Fe, NM: School of American Research Press.

[7] Knight C Studdert-Kennedy M Hurford JR (eds) 2000 The evolutionary emergence of language: social function and the origins of linguistic form. New York, NY: Cambridge University Press.

[8] Stamenov MI Gallese V (eds) 2002 Mirror neurons and the evolution of the brain and language. Amsterdam, The Netherlands: John Benjamins.

[9] Fitch WT 2010 The evolution of language. Amsterdam, The Netherlands: John Benjamins Publishing.

[10] Bolhuis JJ Everaert M (eds) 2013 Birdsong, speech, and language: exploring the evolution of mind and brain. Cambridge, MA: MIT Press.

[11] Sereno MI 1984 ‘DNA’ and language: the nature of the symbolic–representational system in cellular protein synthesis and human language comprehension, p. 346 PhD dissertation, University of Chicago, Chicago, IL, USA.

[12] Sereno MI 1986 A program for the neurobiology of mind. Inquiry 29, 217–240. (10.1080/00201748608602088)

[13] Sereno MI 1991 Four analogies between biological and cultural/linguistic evolution. J. Theor. Biol. 151, 467–507. (10.1016/S0022-5193(05)80366-2) 1943153

[14] Sereno MI 2005 Language origins without the semantic urge. Cogn. Sci. Online 3, 1–12.

[15] Sereno MI 2005 The origin of the human mind: brain imaging and evolution. University of California TV Series: ‘Grey Matters’ See http://www.youtube.com/watch?v=rconzwB422s.

[16] Gentner D 1983 Struture-mapping: a theoretical framework for analogy. Cogn. Sci. 7, 155–170. (10.1207/s15516709cog0702_3)

[17] Judson HF 1979 The eighth day of creation. New York, NY: Simon and Schuster.

[18] Wills C Bada J 2000 The spark of life: Darwin and the primeval soup. Cambridge, MA: Perseus Publishing.

[19] Yoshida M Muneyuki E Hisabori T 2001 ATP synthase: a marvellous rotary engine of the cell. Nat. Rev. Mol. Cell Biol. 2, 669–677. (10.1038/35089509) 11533724

[20] Lewontin RC 1970 The units of selection. Annu. Rev. Ecol. Syst. 1, 1–18. (10.1146/annurev.es.01.110170.000245)

[21] Baldwin JM 1902 Development and evolution. New York, NY: Macmillan.

[22] Mithen S 2005 The singing neanderthals: the origins of music, language, mind, and body. London, UK: Weidenfeld and Nicolson.

[23] Yip M 2013 Structure in human phonology and in birdsong: a phonologist's perspective. In Birdsong, speech, and language: exploring the evolution of mind and brain (eds Bolhuis JJ Everaert M), pp. 181–208. Cambridge, MA: MIT Press.

[24] Gilbert W 1986 Origin of life: the RNA world. Nature 319, 618 (10.1038/319618a0)

[25] Woese CR 1967 The genetic code. New York, NY: Harper and Row.

[26] Crick FH 1968 The origin of the genetic code. J. Mol. Biol. 38, 367–379. (10.1016/0022-2836(68)90392-6) 4887876

[27] Orgel LE 1968 Evolution of the genetic apparatus. J. Mol. Biol. 38, 381–393. (10.1016/0022-2836(68)90393-8) 5718557

[28] Alberts B 2008 Molecular biology of the cell. New York, NY: Garland Science.

[29] Ban N Nissen P Hansen J Moore PB Steitz TA 2000 The complete atomic structure of the large ribosomal subunit at 2.4 A resolution. Science 289, 905–920. (10.1126/science.289.5481.905) 10937989

[30] Nissen P Hansen J Ban N Moore PB Steitz TA 2000 The structural basis of ribosome activity in peptide bond synthesis. Science 289, 920–930. (10.1126/science.289.5481.920) 10937990

[31] Yusupov MM Yusupova GZ Baucom A Lieberman K Earnest TN Cate JHD Noller HF 2001 Crystal structure of the ribosome at 5.5 A resolution. Science 292, 868–899. (10.1126/science.1060089) 11283358

[32] Robertson MP Joyce GF 2012 The origins of the RNA world. Cold Spring Harb. Perspect. Biol. 4, a003608 (10.1101/cshperspect.a003608) 20739415 PMC3331698

[33] Miller SL Orgel LE 1974 The origins of life on earth. Englewood Cliffs, NJ: Prentice-Hall.

[34] Schwartz AW 1998 Origins of the RNA world. In The molecular origins of life (ed. Brack A), pp. 237–254. Cambridge, UK: Cambridge University Press.

[35] Joyce GF Schwartz AW Miller SL Orgel LE 1987 The case for an ancestral genetic system involving simple analogues of the nucleotides. Proc. Natl Acad. Sci. USA 84, 4398–4402. (10.1073/pnas.84.13.4398) 2440020 PMC305096

[36] Illangasekare M Kovalchuke O Yarus M 1997 Essential structure of a self-aminoacylating RNA. J. Mol. Biol. 274, 519–529. (10.1006/jmbi.1997.1414) 9417932

[37] Zhang B Cech TR 1997 Peptide bond formation by *in vitro* selected ribozymes. Nature 390, 96–100. (10.1038/36375) 9363898

[38] Cech TR 2009 Crawling out of the RNA world. Cell 136, 599–602. (10.1016/j.cell.2009.02.002) 19239881

[39] Hauser M 1996 The evolution of communication. Cambridge, MA: MIT Press.

[40] Bickerton D 1990 Language and species. Chicago, IL: University of Chicago Press.

[41] Jackendoff R 1999 Possible stages in the evolution of the language capacity. Trends Cogn. Sci. 3, 272–279. (10.1016/S1364-6613(99)01333-9) 10377542

[42] Zahavi A 1993 The fallacy of conventional signalling. Phil. Trans. R. Soc. Lond. B 340, 227–230. (10.1098/rstb.1993.0061) 8101657

[43] Knight C 2002 Language and revolutionary consciousness. In The transition to language (ed. Wray A), pp. 138–160. Oxford, UK: Oxford University Press.

[44] Cheney DL Seyfarth RM 1990 How monkeys see the world. Chicago, IL: University of Chicago Press.

[45] Shannon CE 1948 A mathematical theory of communication. Bell Syst. Tech. J. 27, 379–423. (10.1002/j.1538-7305.1948.tb01338.x)

[46] Darwin C 1859 On the origin of species. Cambridge, MA: Harvard University Press (facsimile of first edition).

[47] Darwin C 1971 The descent of man, and selection in relation to sex, 2nd edn (revised and augmented). New York, NY: Appleton.

[48] Barrington D 1773 Experiments and observations on the singing of birds. Phil. Trans. R. Soc. Lond. 63, 249–291. (10.1098/rstl.1773.0031)

[49] Konishi M 1985 Birdsong: from behavior to neuron. Annu. Rev. Neurosci. 8, 125–170. (10.1146/annurev.ne.08.030185.001013) 3885827

[50] Nelson DA Marler P 1989 Categorical perception of a natural stimulus continuum: birdsong. Science 244, 976–978. (10.1126/science.2727689) 2727689

[51] Doupe AJ Kuhl PK 1999 Birdsong and human speech: common themes and mechanisms. Annu. Rev. Neurosci. 22, 567–631. (10.1146/annurev.neuro.22.1.567) 10202549

[52] Merker B 2000 Gibbon songs and human music from an evolutionary perspective. In The origins of music (eds Wallin NL Merker B Brown S), pp. 103–124. Cambridge, MA: MIT Press.

[53] Owren MJ Seyfarth RM Cheney DL 1997 The acoustic features of vowel-like grunt calls in chacma baboons (*Papio cynocephalus ursinus*): implications for production processes and functions. J. Acoust. Soc. Am. 101, 2951–2963. (10.1121/1.418523) 9165741

[54] Fitch WT Reby D 2001 The descended larynx is not uniquely human. Proc. R. Soc. Lond. B 268, 1669–1675. (10.1098/rspb.2001.1704) PMC1088793 11506679

[55] Ralls K Fiorelli P Gish S 1985 Vocalizations and vocal mimicry in captive harbor seals, *Phoca vitulina*. Can. J. Zool. 63, 1050–1056. (10.1139/z85-157)

[56] Nottebohm F Stokes TM Leonard CM 1976 Central control of song in the canary, *Serinus canarius*. J. Comp. Neurol. 165, 457–486. (10.1002/cne.901650405) 1262540

[57] Arends JJ Dubbeldam JL 1982 Exteroreceptive and proprioceptive afferents of the trigeminal and facial motor nuclei in the mallard (*Anas platyrhynchos* L.). J. Comp. Neurol. 209, 313–329. (10.1002/cne.902090309) 7130459

[58] Ploog D 1981 Neurobiology of primate audio-vocal behavior. Brain Res. 228, 35–61. (10.1016/0165-0173(81)90011-4) 7023614

[59] Kirzinger A Jurgens U 1991 Vocalization-correlated single-unit activity in the brain stem of the squirrel monkey. Exp. Brain Res. 84, 545–560. (10.1007/BF00230967) 1864326

[60] Simonyan K Jurgens U 2003 Efferent subcortical projections of the laryngeal motorcortex in the rhesus monkey. Brain Res. 974, 43–59. (10.1016/S0006-8993(03)02548-4) 12742623

[61] Gentner TQ Fenn MK Margoliash D Nusbaum HC 2006 Recursive syntactic pattern learning by songbirds. Nature 440, 1204–1207. (10.1038/nature04675) 16641998 PMC2653278

[62] ten Cate C Lachlan R Zuidema W 2013 Analyzing the structure of bird vocalizations and language: finding common ground. In *Birdsong, speech, and language: exploring the evolution of mind and brain* (eds JJ Bolhuis, M Everaert), pp. 243–260. Cambridge, MA: MIT Press.

[63] Zahavi A 1975 Mate selection: a selection for a handicap. J. Theor. Biol. 53, 205–214. (10.1016/0022-5193(75)90111-3) 1195756

[64] Brenowitz EA Arnold AP 1985 Lack of sexual dimorphism in steroid accumulation in vocal control brain regions of duetting song birds. Brain Res. 344, 172–175. (10.1016/0006-8993(85)91205-3) 4041866

[65] Tyack PL Sayigh LS 1997 Vocal learning in cetaceans. In Social influences on vocal development (eds Snowdon CT Hausberger M), pp. 208–233. Cambridge, UK: Cambridge University Press.

[66] Zann R Dunstan E 2008 Mimetic song in superb lyrebirds: species mimicked and mimetic accuracy in different populations and age classes. Anim. Behav. 76, 1043–1054. (10.1016/j.anbehav.2008.05.021)

[67] Eens M 1997 Understanding the complex song of the European starling: an integrated ethological approach. Adv. Study Behav. 26, 355–434. (10.1016/S0065-3454(08)60384-8)

[68] Kendon A 2011 Some modern considerations for thinking about language evolution: a discussion of the evolution of language by tecumseh fitch. Public J. Semiotics 3, 79–108.

[69] Sereno ML Allman JM 1991 Cortical visual areas in mammals. In The neural basis of visual function (ed. Leventhal A), pp. 60–172. London, UK: Macmillan.

[70] Markov NT 2014 A weighted and directed interareal connectivity matrix for macaque cerebral cortex. Cereb. Cortex 24, 17–36. (10.1093/cercor/bhs270) 23010748 PMC3862262

[71] Jackendoff R 1987 Consciousness and the computational mind. Cambridge, MA: MIT Press.

[72] Fauconnier G 1985 Mental spaces. Cambridge, MA: MIT Press.

[73] Lakoff G 1987 Women, fire, and dangerous things. Chicago, IL: University of Chicago Press.

[74] Langacker R 1987 Foundations of cognitive grammar. Stanford, CA: Stanford University Press.

[75] Hasson U Yang E Vallines I Heeger DJ Rubin N 2008 A hierarchy of temporal receptive windows in human cortex. J. Neurosci. 28, 2539–2550. (10.1523/JNEUROSCI.5487-07.2008) 18322098 PMC2556707

[76] Sereno AB Maunsell JH 1998 Shape selectivity in primate lateral intraparietal cortex. Nature 395, 500–503. (10.1038/26752) 9774105

[77] Rubens AB Kertesz A 1983 The localization of lesions in transcortical aphasias. In Localization in neuropsychology (ed. Kertesz A), pp. 245–268. New York, NY: Academic Press.

[78] Burnstine TH Lesser RP Hart J Uematsu S Zinreich SJ Krauss GL Fisher RS Vining EPG Gordon B 1990 Characterization of the basal temporal language area in patients with left temporal lobe epilepsy. Neurology 40, 966–970. (10.1212/WNL.40.6.966) 2345619

[79] Sharp DJ Scott SK Wise RJS 2004 Retrieving meaning after temporal lobe infarction: the role of the basal language area. Ann. Neurol. 56, 836–846. (10.1002/ana.20294) 15514975

[80] Boatman D Gordon B Hart J Selnes O Migioretti D Lenz F 2000 Transcortical sensory aphasia: revisited and revised. Brain 123, 1634–1642. (10.1093/brain/123.8.1634) 10908193

[81] Gross CG 1973 Inferotemporal cortex and vision. Prog. Psychobiol. Physiol. Psych. 5, 77–123.

[82] Potter MC Kroll JF Yachzel B Carpenter E Sherman J 1986 Pictures in sentences: understanding without words. J. Exp. Pychol. Gen. 115, 281–294. (10.1037/0096-3445.115.3.281) 2944988

[83] Lupyan G Ward EJ 2013 Language can boost otherwise unseen objects into visual awareness. Proc. Natl Acad. Sci. USA 110, 14 196–14 201. (10.1073/pnas.1303312110) PMC3761589 23940323

[84] Thompson RL Vinson DP Vigliocco G 2010 The link between form and meaning in British Sign Language: lexical processing effects in a phonological decision task. J. Exp. Psychol. Learn. Mem. Cogn. 36, 1017–1027. (10.1037/a0019339) 20565217

[85] Bosworth RG Emmorey K 2010 Effects of iconicity and semantic relatedness on lexical access in American Sign Language. J. Exp. Psychol. Learn. Mem. Cogn. 36, 1573–1581. (10.1037/a0020934) 20919784 PMC2970771

[86] Klima ES Bellugi U 1979 The signs of language. Cambridge, MA: Harvard University Press.

[87] Finger T 1978 Gustatory pathways in the bullhead catfish II. facial lobe connections. J. Comp. Neurol. 180, 691–706. (10.1002/cne.901800404) 79574

[88] Thompson RL David P Vinson DP Woll B Vigliocco G 2012 The road to language learning is iconic: evidence from British Sign Language. Psychol. Sci. 23, 1443–1448. (10.1177/0956797612459763) 23150275

